# Host Soluble Mediators: Defying the Immunological Inertness of *Aspergillus fumigatus* Conidia

**DOI:** 10.3390/jof4010003

**Published:** 2017-12-24

**Authors:** Sarah Sze Wah Wong, Vishukumar Aimanianda

**Affiliations:** Unité des Aspergillus, Department of Mycology, Institut Pasteur, 75015 Paris, France

**Keywords:** *Aspergillus fumigatus*, innate immunity, humoral immunity, complement

## Abstract

*Aspergillus fumigatus* produce airborne spores (conidia), which are inhaled in abundant quantity. In an immunocompromised population, the host immune system fails to clear the inhaled conidia, which then germinate and invade, leading to pulmonary aspergillosis. In an immunocompetent population, the inhaled conidia are efficiently cleared by the host immune system. Soluble mediators of the innate immunity, that involve the complement system, acute-phase proteins, antimicrobial peptides and cytokines, are often considered to play a complementary role in the defense of the fungal pathogen. In fact, the soluble mediators are essential in achieving an efficient clearance of the dormant conidia, which is the morphotype of the fungus upon inhalation by the host. Importantly, harnessing the host soluble mediators challenges the immunological inertness of the dormant conidia due to the presence of the rodlet and melanin layers. In the review, we summarized the major soluble mediators in the lung that are involved in the recognition of the dormant conidia. This knowledge is essential in the complete understanding of the immune defense against *A. fumigatus*.

## 1. Introduction

*Aspergillus fumigatus* is a ubiquitous fungus that produces spores, called “conidia”, through asexual reproduction [1]. These conidia are small-sized (2–3 µm) and encased with a hydrophobic cell wall and are, therefore, airborne [2]. This opportunistic fungal pathogen can cause a range of infections. For instance, invasive pulmonary aspergillosis under immunocompromised status, chronic pulmonary aspergillosis, and allergic bronchopulmonary aspergillosis in populations with underlying pulmonary dysfunctions [3]. In otherwise healthy populations, the inhaled conidia are efficiently cleared by the immune system and no inflammatory response would be triggered.

Upon inhalation, the conidia (in the dormant morphotype) travel down the airway to reach the lung cavity, where they would be cleared by alveolar macrophages and neutrophils. The recognition of the conidia by the immune cells are challenged by the presence of the rodlet and melanin layers of the outer cell wall of *A. fumigatus* that masks the immunogenic polysaccharides in the inner cell wall [4,5]. The rodlet layer is composed of mainly the hydrophobic protein RodA, whereas melanin layer is present underneath the rodlet layer [6]. As the conidia swell, this rodlet and melanin layers are shed, exposing the polysaccharides in the inner cell wall that contains polysaccharides (β-glucan, α-glucan, chitin, and galactomannan).

However, dormant conidia are phagocytosed despite the shielding of the immunogenic polysaccharides by the rodlet and melanin layers. The immune receptors recognizing fungal cell wall polysaccharides are comparatively well studied, but these cell wall polysaccharides in dormant conidia are not easily accessible to the immune receptors because of the rodlet-melanin layers. The role of soluble mediators in the antifungal defense has been neglected but it is crucial in the efficient clearance of conidia in the early stage of host immune defense as they are the first components to interact with *A. fumigatus* spores before the innate immune cells. Soluble mediators, including complement system, acute-phase proteins, antimicrobial peptides, and cytokines, constitute an integral part of innate immune response [7]. The responsible soluble mediators involved in the immune recognition of dormant *A. fumigatus* conidia are mainly related to complement components and acute-phase proteins, which would be discussed in the following sections. Antimicrobial peptides and cytokines are not involved in the immune recognition of dormant conidia, but rather, contribute to the clearance of conidia by other means. Antimicrobial peptides exert direct antifungal activities on the *A. fumigatus* or enhance the intracellular killing by immune cells [8,9,10], while, cytokines impose pro-inflammatory response by recruiting immune cells or modulating their antifungal activities [8].

This review will summarize the soluble mediators that are known to bind to the dormant conidial surface of *A. fumigatus* and bridge the immune recognition of dormant conidia which challenge their immunological inertness (summarized in Table 1). It is important to elucidate (1) the soluble mediators that bind to the surface of the dormant *A. fumigatus* conidia; (2) to which ligands they are binding; and (3) the immune receptors responsible for the opsonin-mediated recognition.

## 2. Complement Components Associated with Recognition of Dormant Conidia

The complement system is a humoral arm of innate immunity that consists of the classical, lectin, and alternative pathways [11]. The three pathways are activated by the binding of distinct pattern-recognition molecules on the pathogen surface, forming complement component C3 convertase. The alternative pathway starts with the auto-hydrolysis of C3 into C3b. Immunoglobulin has also been demonstrated to activate alternative pathway [12]. Classical pathway is antibody-mediated that starts with the binding of IgM or IgG on the microbial surface. The lectin pathway is activated by the binding of mannose-binding lectin or ficolin, that interact with serine proteases (MASPs), on the pathogen surface [13]. The C3 convertase cleaves C3 into opsonins C3b or iC3b (inactivated form of C3b that is incapable of amplifying). The cascade then continues to achieve the three main ultimate goals, as follows: (1) opsonization by C3b and iC3b; (2) recruitment of immune cells by production of anaphalytoxins C3a and C5a; and (3) direct lytic killing of the pathogen by formation of membrane attack complex (MAC). However, due to the presence of a thick fungal cell wall, the MAC is unlikely to kill the fungi by membrane lysis; therefore, the importance of complement system on antifungal defense has always been overlooked. In fact, the complement system is crucial in enhancing immune recognition of the conidia via opsonization [14], in particular, for the dormant conidia. 

Many studies have demonstrated that dormant conidia activate mainly the alternative pathway, while there is a growing activation of classical pathway as the conidia swell and germinate [15,16,17]. The major complement component C3 has been shown to bind to the surface of *A. fumigatus* conidia and mycelia [15,18]. The surface bound C3 is rapidly converted to the inactivated form iC3b [18]. iC3b is the primary form deposited on dormant conidia while there is a mixture for both on swollen and germinating conidia [15,18]. iC3b is incapable of the formation of C3 convertase and, thus, the amplification of C3b is halted [11]. 

Mannose-binding lectin (MBL) is C-type lectin (collectin) that binds to mannose via its carbohydrate recognition domain in a calcium-dependent manner [19]. MBL binds to dormant *A. fumigatus* conidia [16,20]. Ficolin (fibrinogen + collagen + lectin) is another family of lectin that consists of a collagenous domain and a fibrinogen domain that recognizes *N*-acetylglucosamine (GlcNAc) [21]. There are three ficolins (ficolin-1, ficolin-2, and ficolin-3) in human [21]. Ficolin-2 and ficolin-3 bind to dormant *A. fumigatus* conidia in a calcium-independent manner [22,23]. Normally, in the lectin pathway, MASPs form complexes with MBL or ficolins, and cleaves C4 to form the C3 convertase (C4bC2a) and, thus, activating C3. However, it has been shown that the MBL bound to *A. fumigatus* conidia directly activate C3 without the formation of C4bC2a [16]. Similar to MBL, ficolin-2 opsonisation of conidia does not lead to C4 deposition, suggesting the absence of lectin pathway activation [24]. Taken together, the lectin pathway is not activated by dormant conidia. Instead, the MBL-MASP and/or ficolin-MASP complexes bound on the conidial surface activate C3 for opsonisation. In fact, MASP can directly activate C3 [11].

The classical pathway is activated by the binding of C1 complex (consisting of C1q, C1r, and C1s) to immunoglobulin that bind to the surface antigen of the pathogen [11]. C1q was also shown to bind to dormant conidia in the presence of serum, which leads to activation of C3 [25,26]. 

It raises the question of the identity of the ligand(s) to which MBL, ficolins and C1q bind. It was shown that melanin of *Cryptoccocus neoformans* and *Aspergillus niger* activate the alternative pathway [27], suggesting that melanin may not be the ligand for MBL, ficolins, and C1q binding. The melanin in *C. neoformans* and *A. niger* is synthesized through the DOPA (3,4-dihydroxyphenylalanine) pathway [28,29], while that of *A. fumigatus* is through the DHN (1,8-dihydroxynaphthalene) pathway [30]. Therefore, it is worth checking the role of DHN melanin in complement activation.

## 3. Non-Complement Components Associated with the Recognition of Dormant Conidia

In addition to the complement components, the acute-phase proteins (APPs) make up another group of soluble mediators. The acute-phase proteins (APPs) are defined as the proteins with significant changes in plasma concentrations in response to the events of infection, inflammation or tissue injury [31]. Cytokines, C-reactive proteins and several complement proteins, for instance, C3, C4, and mannose-binding lectin, are also acute-phase proteins [31]. 

The lung surfactant proteins A and D (SP-A and SP-D) are collectins (calcium-dependent lectins consisting of collagenous region and a carbohydrate recognition domain) that play a major role in pulmonary immunity [32]. Although they are not classical APPs, the levels of SP-A and SP-D increase following infection or inflammation, for instance, following the inoculation of intratracheal lipopolysaccharide [33], and following allergic airway challenge of *A. fumigatus* [34]. SP-A and SP-D are crucial to the immune defense against *A. fumigatus* [35]. They bind, with dependency to calcium, to dormant *A. fumigatus* conidia and facilitate phagocytosis and killing by neutrophils and alveolar macrophages [36]. Then, later, another study contrastingly demonstrated that the binding of SP-A and SP-D to dormant *A. fumigatus* conidia is independent of calcium [37]. This discrepancy leaves the identity of the responsible ligand(s) unclear.

Other recently-discovered collectins, namely, collectin-11 (CL-K1) and collectin-12 (CL-P1), are also demonstrated to bind *A. fumigatus* conidia in a calcium-dependent manner [38,39]. Similarly to other collectins, the binding of CL-K1 does not lead to complement activation [38]. 

The pentraxin-related protein 3 (PTX3) is a member of the pentraxin family, which is a pattern-recognition receptor and acute-phase proteins. Ptx3 binds to *A. fumigatus* conidia through the *N*-terminal domain, while the *C*-terminal domain is required for receptor binding [40]. Ptx3-deficient mice are susceptible to invasive aspergillosis [41]. Ptx3 also binds to C1q [42]. Administration of PTX3 to C1q-deficient mice reverses the susceptibility, proving the non-redundant role of PTX3 independent of C1q [41].

A high level of C-reactive protein (CRP) is demonstrated in invasive aspergillosis patients [43,44]. CRP is similar to immunoglobulin G as they both activate the complement cascade and are recognized by the Fcγ receptors [45]. CRP binds to *A. fumigatus* hyphal fractions [46] and enhances neutrophil phagocytosis of dormant conidia through opsonization [47]. Apart from this information, nothing else is known about the interaction between CRP and dormant *A. fumigatus* conidia, which could not be ignored.

## 4. Immune Receptors

Following the binding of soluble mediators to the conidia, the immune-complex binds to receptors on immune cells to trigger phagocytosis and inflammatory response. The relationship between soluble mediators and immune receptors are not exclusive, but rather, promiscuous. In fact, it is a complex network where one soluble mediator can be recognized by several immune receptors, and vice versa.

### 4.1. Complement Receptors and Fcγ Receptors

The major types of opsonic receptors found on phagocytic cells are Fcγ receptors (FcγRs) and complement receptors (CR1, CR3, and CR4).

FcγRs recognize dormant conidia that are opsonized by IgG or CRP and, thus, promote phagocytosis [45]. Complement receptor 3 (CR3) and complement receptor 4 (CR4) are heterodimeric integrins that share a common β_2_ subunit (CD18) and have different α subunits. The α subunits for CR3 and CR4 are α_M_ (CD11b) and α_x_ (CD11c), respectively. CR3 recognizes a broad range of ligands, including iC3b, β-1,3-glucan, Ptx3, and LPS [40,48], while CR4 mainly recognizes iC3b [49]. Although the α subunits of CR3 and CR4 are highly similar, and both CR3 and CR4 bind iC3b, the binding sites involved on iC3b for binding to the two CRs are different [50]. In addition, the structure of the cytoplasmic domains of the two CRs are different which may contribute to different downstream signaling pathways [51]. CR3-mediated phagocytosis requires the activation of Rho which then induces actin polymerization [52]. On the other hand, it has shown that CR4 mediates the binding to iC3b-opsonized particles, but the phagocytosis requires the participation of CR3 [53]. Complement receptor 1 (CR1) (CD35) recognizes C3b, C1q, and MBL and mediates phagocytosis of the opsonized particle by activating phospholipase D [54,55,56]. Importantly, the adhesion and phagocytosis of iC3b- or C3b-opsonized particles mediated by complement receptors alone do not trigger the production of respiratory burst, which is required for the intracellular killing of the internalized conidia [57,58]. The production of respiratory burst requires the cooperation between CR3 and FcγRIII [59].

The three main complement receptors also differ in their amount in various cell types [51]. For instance, CR4 is highly expressed on tissue macrophages; in contrast, CR3 and CR1 are slightly expressed. CR3 and CR1 are highly expressed on neutrophils, but CR4 is otherwise slightly expressed. This renders CR4 to have a significant role in the recognition of *A. fumigatus* conidia, as the alveolar macrophages are the first type of professional phagocytes to encounter the inhaled conidia before the neutrophils. However, the importance of CR4 is unclear as most of the phagocytosis could be largely inhibited by blocking CR3 alone by antibodies [51]. Little is known about the role of these four receptors in the phagocytosis of dormant *A. fumigatus* conidia. Only one study that has contributed to the knowledge that the long pentraxin 3 (Ptx3) opsonizes *A. fumigatus* conidia and facilitates phagocytosis via the FcγR and CR3 [40].

### 4.2. Calreticulin

The recognition of the other opsonins, besides iC3b and C3b, of dormant *A. fumigatus* conidia (C1q, SP-A, SP-D, and MBL) is mediated by the calreticulin-CD91 complex [60,61]. Calreticulin is an intracellular chaperone that lacks a transmembrane domain. By binding to CD91 on macrophage surface, the calreticulin-CD91 complex is able to mediate phagocytic uptake [60]. It was previously believed that calreticulin binds to the collagen-like region of collectins, while the CD91 acts as a transmembrane anchor that then activates signaling transduction for initiating phagocytosis. However, it was later found that CD91 also directly binds to C1q. In addition, the signaling transduction of phagocytosis by the calreticulin-CD91 complex remained unidentified. SP-A and SP-D bind to calreticulin/CD91 via the collagenous region to initiate a pro-inflammatory response, while they bind SIRPα via the globular head [62].

## 5. Conclusions

Soluble mediators of innate immunity are crucial in the recognition of dormant *A. fumigatus*, which has a sophisticated mechanism of masking its immunogenic cell wall polysaccharides by the rodlet and melanin layers. Here in this review, we have summarized the responsible soluble mediators, for which most of them are collectins. Importantly, the ligands for collectin binding remain unidentified. Further investigation of the interaction between these aforementioned collectins and the major components of the rodlet layer, RodA protein, and melanin, should be warranted. It would be worthy to examine all the remaining collectins of their ability to recognize dormant *A. fumigatus* conidia. Surprisingly, the role of immune receptors in the recognition of dormant *A. fumigatus* conidia opsonized by soluble mediators remained unexplored.

## Figures and Tables

**Table 1:** Soluble mediators interacting with *A. fumigatus* conidia, responsive receptors on effector immune cells involved in their recognition and their proven or possible function.

Soluble Mediator	Immune Receptor	Effective Immune Cells	Function	Reference
C3: C3b/iC3b	CR1, CR3, CR4	Macrophages, dendritic cells, neutrophils, monocytes	C3b and iC3b opsonizes conidia facilitating phagocytosis Opsonization interferes with dissemination of *A. fumigatus* (antimicrobial effect)	[14,15,17,18]
C3: C3d	CR2 (CD35)	B cells	C3d-opsonized conidia lowers threshold for B cell activation	[14]
C1q	C1qR	Monocytes, polymorphonuclear leukocytes	Interaction with Ptx3 activates classical pathway on *A. fumigatus* conidia	[63]
Ficolin-2 and Ficolin-3	Their interaction with the immune cells is rather indirect, through activation of lectin pathway		Form complex with MASPs, activating C3 Ficolin-2 interacts with Ptx3, activating lectin pathway MBL has been shown to activate C3 directly Ficolin-3 opsonizes conidia and activates lectin pathway	[23,64]
Mannose-binding lectin (MBL)				[16,65]
C-reactive protein	FcγR	Neutrophils	Opsonization and phagocytosis	[45,47]
Surfactant proteins A and D (SP-A & SP-D)	Calreticulin-CD91 complex, C1qR	Neutrophils, alveolar macrophages	Opsonization and conidial agglutination	[36]
Collectins 11 and 12 (CL-K1 & CL-P1)	No direct binding, but through complement activation		Binding and activation of lectin complement pathway	[38,39]
Pentraxin, Ptx3	Through FcγR and CR3	Neutrophils	Opsonization and activation of alternative pathway Interaction with C1q or ficolin-2 to activate classical and lectin pathways, respectively, thus leading to conidial opsonization by C3b	[14,40,64]
